# Mulberry Leaf Dietary Supplementation Can Improve the Lipo-Nutritional Quality of Pork and Regulate Gut Microbiota in Pigs: A Comprehensive Multi-Omics Analysis

**DOI:** 10.3390/ani14081233

**Published:** 2024-04-19

**Authors:** Junjie Hou, Xiang Ji, Xiaoran Chu, Binjie Wang, Kangle Sun, Haibo Wei, Yu Zhang, Zhen Song, Fengyun Wen

**Affiliations:** 1 College of Animal Scienceand Technology, Henan University of Science and Technology, Luoyang 471003, China; houjunjie@stu.haust.edu.cn (J.H.);; 2 The Kay Laboratory of High Quality Livestock and Poultry Germplasm Resources and Genetic Breeding of Luoyang, College of Animal Science and Technology, Henan University of Science and Technology, Luoyang 471003, China

**Keywords:** lipidomic, intramuscular fat, mulberry leaves, triglyceride, lipid profile

## Abstract

**Simple Summary:**

Regulating the lipid nutritional profile of meat by balancing intramuscular fat and backfat is a difficult problem in pork production. In this study, we found that feed supplementation with mulberry leaves increased intramuscular fat while simultaneously reducing backfat. The results of our study suggest that nutritional supplementation with mulberry leaves may be used in pork production to improve the lipid nutrition of meat.

**Abstract:**

Mulberry leaves, a common traditional Chinese medicine, represent a potential nutritional strategy to improve the fat profile, also known as the lipo-nutrition, of pork. However, the effects of mulberry leaves on pork lipo-nutrition and the microorganisms and metabolites in the porcine gut remain unclear. In this study, multi-omics analysis was employed in a Yuxi black pig animal model to explore the possible regulatory mechanism of mulberry leaves on pork quality. Sixty Yuxi black pigs were divided into two groups: the control group (*n* = 15) was fed a standard diet, and the experimental group (*n* = 45) was fed a diet supplemented with 8% mulberry leaves. Experiments were performed in three replicates (*n* = 15 per replicate); the two diets were ensured to be nutritionally balanced, and the feeding period was 120 days. The results showed that pigs receiving the diet supplemented with mulberry leaves had significantly reduced backfat thickness (*p* < 0.05) and increased intramuscular fat (IMF) content (*p* < 0.05) compared with pigs receiving the standard diet. Lipidomics analysis showed that mulberry leaves improved the lipid profile composition and increased the proportion of triglycerides (TGs). Interestingly, the IMF content was positively correlated with acyl C18:2 and negatively correlated with C18:1 of differential TGs. In addition, the cecal microbiological analysis showed that mulberry leaves could increase the abundance of bacteria such as UCG-005, *Muribaculaceae*_norank, *Prevotellaceae*_NK3B31_group, and *Limosilactobacillus*. Simultaneously, the relative levels of L-tyrosine-ethyl ester, oleic acid methyl ester, 21-deoxycortisol, N-acetyldihydrosphingosine, and mulberrin were increased. Furthermore, we found that mulberry leaf supplementation significantly increased the mRNA expression of lipoprotein lipase, fatty acid-binding protein 4, and peroxisome proliferators-activated receptor γ in muscle (*p* < 0.01). Mulberry leaf supplementation significantly increased the mRNA expression of diacylglycerol acyltransferase 1 (*p* < 0.05) while significantly decreasing the expression of acetyl CoA carboxylase in backfat (*p* < 0.05). Furthermore, mulberry leaf supplementation significantly upregulated the mRNA expression of hormone-sensitive triglyceride lipase and peroxisome proliferator-activated receptor α (*p* < 0.05) in backfat. In addition, mulberry leaf supplementation led to increased serum leptin and adiponectin (*p* < 0.01). Collectively, this omic profile is consistent with an increased ratio of IMF to backfat in the pig model.

## 1. Introduction

Pork is the most widely produced and consumed meat product worldwide, and it is the main source of protein intake for humans because of its rich and balanced nutrition [1,2]. The quality and safety of pork are closely related to people’s lives. With the global rise of large-scale swine farms, although the production of pork has been secured, the taste and quality of pork have been continuously decreasing [3,4]. Intramuscular fat (IMF) is an important indicator of pork quality and nutrition, as the IMF content is closely related to pork flavor, juiciness, and tenderness [5]; however, IMF has not attracted sufficient attention from the pig farm industry. With the increasing demand for higher-quality meat, the IMF content of meat has gradually increased [6]. However, higher IMF content is often accompanied by greater backfat thickness, and excessive backfat detrimentally affects the lean meat percentage. Therefore, balancing IMF and backfat is an important issue in pork production [7].

Improving feed formulations is currently one of the mainstream strategies for enhancing meat quality [8]. In many countries, the shortage of raw feed materials and continuously improving standards for meat quality are the main inhibitors to the development of the pork breeding industry [9]. Therefore, finding a raw feed material that can not only improve meat quality, but can also be used as a regular feed substitute, is a major research focus of the livestock industry. Mulberry leaf plants of the genus *Morusare* are widely distributed in northwest and south China, Japan, Europe, and Southeast Asia, among other regions [10]. Mulberry leaves have a high protein content (25–30% by mass), are rich in a variety of vitamins and amino acids, and also contain mulberry leaf polysaccharide (MLP). Furthermore, mulberry leaves are widely used in Asia as a protein source in livestock feed [11]. Prior studies have shown that 8–10% mulberry leaf supplementation can effectively improve pork quality [12,13]. Additionally, studies have shown that mulberry leaves can reduce blood lipids while improving blood pressure, immunity, and inflammation; therefore, they are often used as a traditional Chinese medicine or functional food [14,15]. The Yuxi black pig, a breed native to central China, has roughage resistance, disease resistance, and good meat quality. Preliminary evidence shows that mulberry leaves can significantly improve the quality of Yuxi black pig meat and reduce backfat ketone bodies, but the underlying mechanism is unclear [13,16].

Lipidomics is an emerging omics technology used to explore the composition of lipid profiles. Furthermore, lipidomics plays an important role in exploring the regulatory mechanisms of lipid metabolism by screening, classifying, and refining differentially expressed lipid molecules. The gut microbiota refers to the assemblage of microorganisms in the gut. Alterations in the composition of these gut microorganisms can affect the physiology, immunity, and nutrient absorption of the host. Conversely, changes in the health status of the host can lead to corresponding changes in gut microbiota composition [17]. Exploring the relationship between gut microbiota, gut metabolites, and lipid metabolism represents an important current research topic [18,19]. Metabolomics techniques based on high-performance gas chromatography–mass spectrometry are becoming a common tool for the analysis of volatile and non-volatile compounds and are widely used in the identification of food products and the analysis of metabolites for food product characterization [20].

In this study, the effect of mulberry leaves on IMF was explored through comprehensive multi-omics analysis. We also investigated the relationship between differential lipid molecules, gut microbiota, and gut metabolites to identify those that may have an influence on IMF. Finally, we investigated the relative differences in the expression of important regulatory genes in lipid metabolism to identify potential regulatory mechanisms.

## 2. Materials and Methods

### 2.1. Ethics Approval

The animal study was approved by the Institutional Animal Ethics Committee of Henan University of Science and Technology (Approval No. 2022-02-023).

### 2.2. Preparation of Animals and Samples

All procedures involving animals were performed in accordance with the Animal Protection and Welfare Guidelines of the Ministry of Agriculture of China. Yuxi black pigs, aged 90–100 days, were purchased from Luoyang Luanchuan Farming Co., Ltd. (Luoyang, China) with an equal number of males and females, each with an initial weight of approximately 40 kg. Sixty Yuxi black pigs were divided into two groups: the control group (*n* = 15; 7 females, 8 males) was fed a standard diet, and the experimental group (*n* = 45; 23 females, 22 males) was fed a diet supplemented with 8% mulberry leaves. Experiments were performed in three replicates (*n* = 15 per replicate); the two diets were ensured to be nutritionally balanced, and the feeding period was 120 days, following a pre-experimentation acclimation period of 7 days. Table S1 shows the nutrient composition of the feed. Mulberry leaves were purchased from the Henan Sangbio Agricultural Company. After 120 days of feeding, the animals were weighed and fasted for 1 day before slaughter, and approximately 200 g of longissimus thoracis (LT) and backfat tissue at the fifth to sixth rib was collected, along with the cecum and cecal contents.

### 2.3. Determination of Meat Quality

Body weight was determined prior to slaughter, followed by backfat thickness, marbling score, pH, meat color, IMF content, and the triglyceride (TG) content of muscle and backfat. The average backfat thickness of the first rib, last lumbar spine, and last rib was used to define the backfat thickness. The marbling score was determined from a cross-sectional slice of the muscle at the first lumbar spine and ranged from 0 to 5, with higher scores indicating a more abundant IMF content. The color of the LT was determined using OPTO-STAR (MATTHAUS, Berlin, Germany), and the meat color parameters included L* (lightness), a* (intensity), and b* (yellowness).

The pH value of the LT was determined 40–50 min and 24 h after slaughter (HI99163, HANNA, Padua, Italy). The IMF content was measured using a Soxhlete extractor (SX-360, OPSIS, Lund, Sweden). Triglyceride content was determined using a triglyceride kit (EY-01H2206, Shanghai, China), following the manufacturer’s instructions.

### 2.4. Lipidomics Analysis

Thirty milligrams of the sample (the fat-rich part of the LT muscle) was weighed and homogenized by adding an internal standard (Lyso PC-17:0) and 280 μL of a methanol and water solution (*v*/*v* = 1:1) for 2 min (60 Hz), followed by vortexing with chloroform for 30 s and centrifugation for 10 min (12,000× *g*, 4 °C). The lower chloroform layer was then removed, after which 280 μL of a chloroform and methanol solution (*v*/*v* = 2:1) was added to the sample and swirled for 30 s. Next, the mixture was subjected to ultrasonic extraction for 20 min, allowed to rest for 20 min, centrifuged for 10 min (12,000× *g*, 4 °C) to extract lipids, and redissolved with 280 μL of an isopropanol and methanol solution. After further centrifugation for 15 min (12,000× *g*, 4 °C), 150 μL of the supernatant was loaded into a lined LC-MS injection vial for LC-MS analysis.

The column temperature was set to 55 °C. Mobile phase A consisted of acetonitrile and water at a ratio of 6:4 (*v*/*v*), while mobile phase B consisted of isopropanol and acetonitrile at a ratio of 9:1 (*v*/*v*). The flow rate was maintained at 0.26 mL/min, with an injection volume of 2 µL in positive ion mode and 2 μL in negative ion mode, set to a total of 4 μL. The samples were separated using a Dionex U3000 ultra-high-performance liquid chromatograph (Thermo Fisher Scientific™, Waltham, America) and analyzed through mass spectrometry using a Q Exactive Plus (Thermo Scientific™). The detection employed heated electrospray ionization operating in both positive and negative ion modes.

### 2.5. Total RNA Extraction and qPCR

TRIzol reagent (Servicebio, Wuhan, China) was used to extract total RNA from subcutaneous fat and IMF, and a spectrophotometer (NanoDrop 2000, Thermo Fisher Scientific™, Waltham, America) was used to determine the absorbance of RNA at 260 and 280 nm. The RNA was then reverse-transcribed using the SweScript RT I First Strand cDNA Synthesis Kit (Servicebio, China) and random primers. qPCR was performed using a Bio-Rad CFX Connect quantitative PCR fluorescence instrument with an SYBR Green qPCR Master Mix and gene-specific primers (Table S2), and relative expression was calculated using the 2^−ΔΔCt^ method [21].

### 2.6. H&E Staining, Oil Red Staining, and BODIPY Staining

H&E staining: Backfat tissue samples were placed in fixative for 1 day, embedded in paraffin, and made into 10 μm frozen sections. These sections were sequentially immersed in xylene I and II for 15 min, and then sequentially immersed in 95%, 85%, and 70% ethanol for 5 min and then in distilled water for 5 min. An eosin staining solution was added to the tissue sample sections for 3 min to allow the tissue to be fully stained. Then, the slices were sequentially placed in 95% alcohol I, 95% alcohol II, absolute alcohol I, absolute alcohol II, xylene I, and xylene II for 5 min, after which they were dried and sealed with neutral glue. Finally, they were observed under a microscope.

Oil Red O staining: The muscle tissue sections were fixed in 4% paraformaldehyde at 37 °C for 20–30 min, washed with a 70% ethanol solution for 5 s, stained with Oil Red O staining solution for 5–10 min, and then washed with a 70% ethanol solution to remove the excess staining solution. After this, the sections were washed with precooled phosphate-buffered saline (PBS) three times, immersed in hematoxylin solution for 2 min, rinsed with PBS for another 30 s, and finally observed under a microscope.

BODIPY staining: Muscle tissue sections were fixed with 4% formaldehyde or 4% acetaldehyde and then incubated with BODIPY for 30 min. Stained sections were washed three times with PBS or other buffers before being photographed using a fluorescence microscope.

### 2.7. DNA Extraction and 16SrRNA Gene Amplification and Sequencing

DNA extraction from the cecal contents was performed using the Intestinal Fecal DNA Extraction Kit Omega D4015-02 (Omega Bio-Tek, Beijing, China). Subsequent PCR amplification was performed using TransStart FastPfu DNA Polymerase (TransGen AP221-02, Beijing, China) with specific primers and barcodes. The PCR products were quantified using a QuantiFluor™-ST Blue fluorescent Quantitative System (Promega, Madison, America), followed by Illumina PE250 library construction and Illumina PE250 sequencing. The PE reads obtained by Illumina PE250 sequencing were assembled according to the overlap relationship, alongside simultaneous quality control and filtering of the sequence. After distinguishing the samples, operational taxonomic unit (OTU) cluster and taxonomic analyses were performed. The sequencing depth was determined based on taxonomic information, and statistical analyses of the community structure were performed at each taxonomic level.

### 2.8. Untargeted Metabolomics Assays

Relevant experimental methods and procedures for metabolomics performed upon cecal contents can be found in previous studies [22,23].

### 2.9. Statistical Analysis

The experimental data are expressed as the mean ± standard deviation (SD) of three independent experiments. Statistical analyses were performed using SPSS 22.0. GraphPad software (9.0) and Origin 2022 were used for correlation analysis and data visualization, and *p* values less than 0.05 were considered to indicate statistical significance.

## 3. Results

### 3.1. Effect of Mulberry Leaves on Growth Performance and Meat Quality

**Figure 1:**
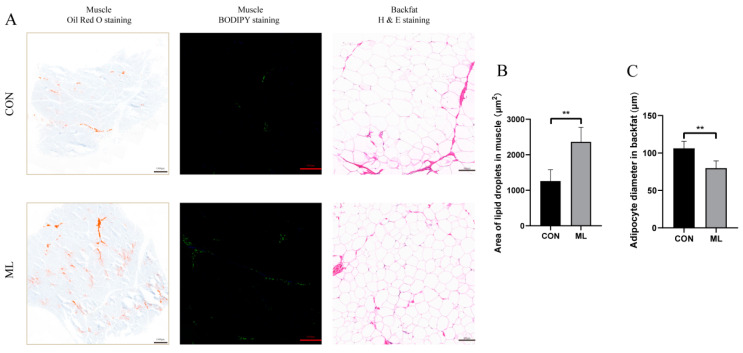
HE staining, Oil Red O staining, and BODIPY staining. (**A**) Oil Red O staining (scale bar: 1000 μm), BODIPY staining (scale bar: 1000 μm) of muscle tissue, and H&E staining (scale bar: 100 μm) of backfat. (**B**) Determination of lipid droplet area in the muscle. (**C**) Determination of the diameter of adipocytes in backfat. ** *p* < 0.01.

The implementation of a diet supplemented with 8% mulberry leaves did not affect the growth performance of Yuxi black pigs (Table 1). Meat quality measurements showed that the marbling score and lightness (L*) of muscle tissue in the mulberry leaf (ML) group increased significantly, whereas pH, shear force, cleavage (a*), and yellowness (b*) were not significantly changed. In addition, pigs fed with mulberry leaves had significantly increased IMF and TG in muscle, as well as significantly reduced backfat thickness and TG in backfat (*p* < 0.05) (Table 2). The Oil red O staining and BODIPY staining of muscle tissue demonstrated that the fat content of muscle in the ML group was significantly higher than that in the control (CON) group (*p* < 0.01) (Figure 1A). The H&E staining of backfat showed that adipocytes were significantly smaller in the ML group than in the CON group (*p* < 0.01) (Figure 1A). These results suggest that feeding Yuxi black pigs a diet supplemented with 8% mulberry leaves could improve their IMF and reduce their backfat thickness.

### 3.2. Distribution of Lipid Profiles of Muscle Tissue and Screening of Differential Lipid Molecules

**Figure 2:**
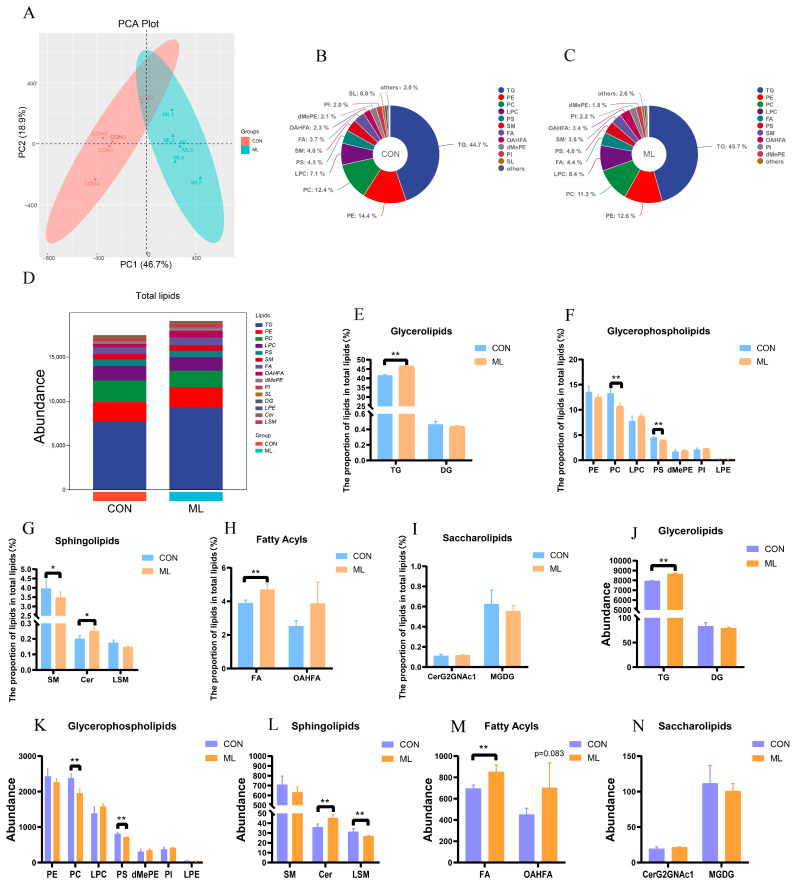
Effect of mulberry leaves on muscle lipid profile composition. (**A**) PCA. (**B**,**C**) Proportions of different lipid species in the CON and ML groups. (**D**) Abundances of different lipid species in the CON and ML groups. (**E**–**I**) Proportions of glycerolipids (**E**), glycerophospholipids (**F**), sphingolipids (**G**), fatty acids (**H**), and saccharolipids (I) in the CON and ML groups. (**J**–**N**) Abundance of glycerolipids (**J**), glycerophospholipids (**K**), sphingolipids (**L**), fatty acids (**M**), and saccharolipids (**N**) in the CON and ML groups. * *p* < 0.05 and ** *p* < 0.01.

The significant differences in IMF quality were further explored by analyzing the distribution of lipids throughout the tissue samples. Principal component analysis (PCA) showed significant differences in lipid profiles between the CON and ML groups (Figure 2A). The lipid compositions of the CON and ML groups are shown in Figure 2B–D. The lipid profiling results showed that the proportions of TG, Cer, and FA in the ML group were significantly higher than those in the CON group, whereas the proportions of PC, PS, and SM were significantly lower than those in the CON group (Figure 2E–I). In addition, the abundances of TG, Cer, and FA in the ML group were significantly higher in the ML group than in the CON group, whereas the abundances of PC, PS, and LSM were significantly lower (Figure 2J–N).

**Figure 3:**
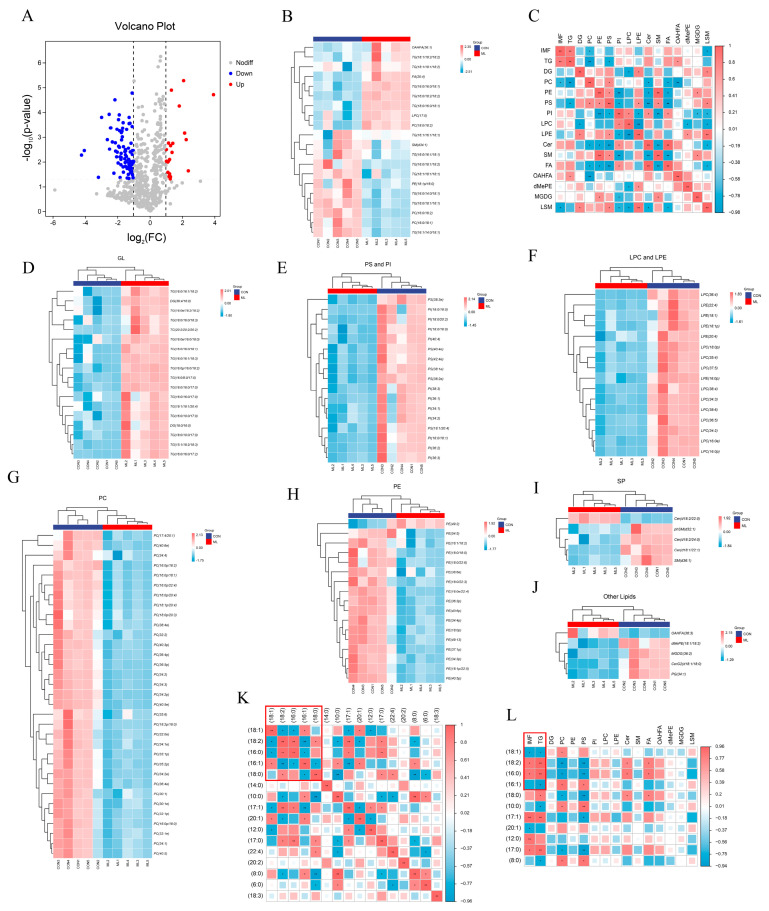
Effects of mulberry leaves on muscle lipid molecules. (**A**) Volcano plots of lipid molecules. (**B**) Heat map of top 20 differentially expressed lipid molecules. (**C**) Heat map of correlations for different lipid species. (**D**–**J**) Heat maps of (**D**) GL, (**E**) PS and PI, (**F**) LPC and LPE, (**G**) PC, (**H**) PE, (**I**) SP, and (**J**) other lipids in the CON and ML groups. (**K**) Heat map of the correlation between the acyl groups of TG. (**L**) Heat map of the correlation between the acyl groups of TG, IMF, and lipid species. * *p* < 0.05 and ** *p* < 0.01.

A total of 777 lipid molecules were identified, and 110 differential lipid molecules (*p* < 0.05, FC > 2) were screened (Figure 3A). A heat map of the top 20 differentially expressed lipid molecules showed that compared with the CON group, TG (18:1/18:2/18:2), TG (18:0/16:0/18:1), and TG (16:0/18:2/18:2) showed an upward trend, whereas TG (16:0/14:0/18:1), TG (18:0/18:1/18:1), and TG (18:1/18:1/18:1) showed a downward trend in the ML group (Figure 3B). Correlation analysis of different lipid species showed that IMF was significantly positively correlated with TG but negatively correlated with PC and LSM (Figure 3C). The differential lipid molecules were classified, and the resulting heat map showed that the major lipid molecules with significantly upregulated expression were GL, PS, PI, LPC, LPE, and SP. In contrast, the major lipid molecules with significantly downregulated expression were PC and PE (Figure 3D–J).

Since TG was the most abundant lipid molecule with significant differences between groups, it was further explored using correlation analysis. The number of carbon atoms in the acyl chain of TG is generally between 4 and 26, with (18:1), (18:2), (16:0), and (16:1) being the most important acyl chains, accounting for more than 70% of them. Correlation analysis of the major acyl chains of TG in the samples revealed that (18:1) was negatively correlated with (18:2) and (16:0) and positively correlated with (16:1), (18:2) was positively correlated with (16:0), and (16:0) was negatively correlated with (16:0) (Figure 3K). Applying correlation analysis to the acyl chains and IMF content of samples demonstrated that IMF was positively correlated with (18:2) and (16:0) and negatively correlated with (18:1) and (16:1) (Figure 3L).

In summary, feeding pigs a diet supplemented with 8% mulberry leaves improved the lipid profile of muscle mainly by increasing TG levels, among which TG (16:0/16:0/18:1), TG (18:0/16:0/18:1), and TG (18:0/16:0/18:1) were the main lipid molecules with upregulated expression. Additionally, IMF content was positively correlated with C18:2 in TG and negatively correlated with C18:1 in TG.

### 3.3. Effects of Mulberry Leaves on Gut Microbiota

**Figure 4:**
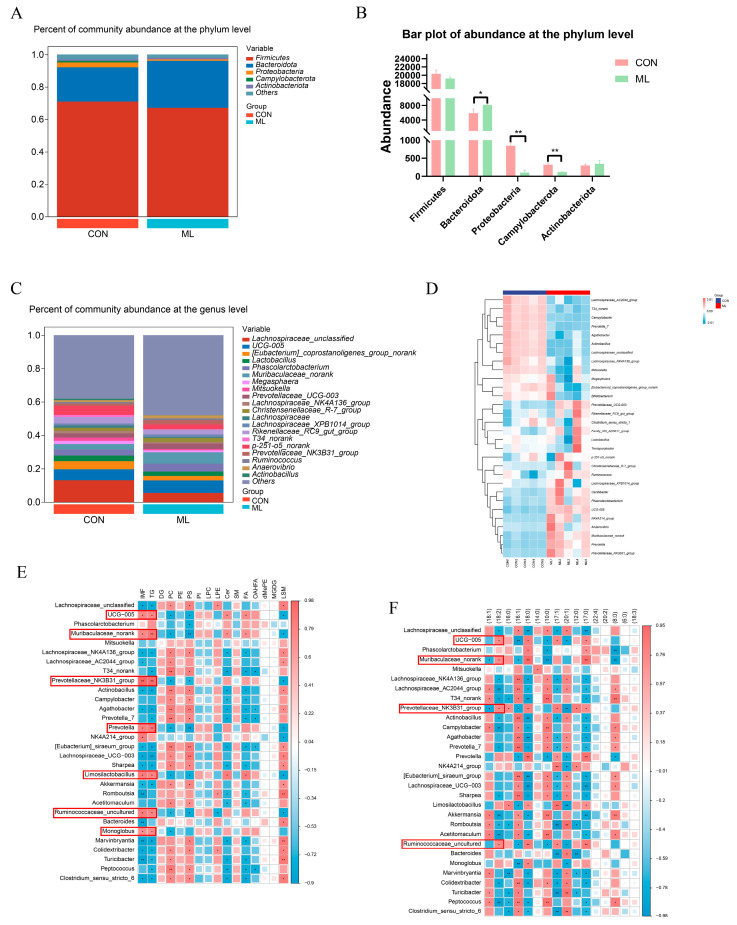
Effects of mulberry leaves on cecum microbiota. (**A**) The proportion of bacteria at the phylum level. (**B**) Bacterial abundance at the phylum level. (**C**) The proportion of bacteria at the genus level. (**D**) Heat map of the top 30 most abundant bacteria. (**E**) Heat map showing the correlation between the differential bacteria and lipid species. (**F**) Heat map of the correlation between the differential bacteria and acyl groups of TG. * *p* < 0.05 and ** *p* < 0.01.

To explore the effects of mulberry leaves on the cecal bacterial composition in Yuxi black pigs, cecal bacterial diversity was analyzed. We detected and screened 720,798 sequences. Through OTU analysis of non-repetitive sequences with 97% similarity, 4496 OTUs were obtained, and the rarefaction curve tended to be flat (Figure S1). The results of the diversity analysis of colony composition showed that the Chao1 index, Shannon index, and Simpson index were not significantly different between the CON and ML groups (Figure S2). At the phylum level, the abundance of Bacteroidetes was significantly increased (*p* < 0.05). The abundances of Proteobacteria and Campylobacterota were significantly decreased (*p* < 0.01). In contrast, the abundances of Firmicutes and Actinobacteria were not significantly changed (Figure 4B). At the genus level, the top 20 bacterial genera in the CON and ML groups were plotted in stacked bar graphs, as shown in Figure 4C. The top five were *Lachnospiraceae*_unclassified, UCG-005, [*Eubacterium*]_*coprostanoligenes*_group_norank, *Lactobacillus*, and *Phascolarctobacterium*. Subsequently, a heat map was drawn for the top 30 differential bacteria, which revealed that the abundances of UCG-005, *Muribaculaceae*_norank, and *Prevotella* showed an increasing trend, whereas the abundances of *Campylobacter*, *Prevotella*_7, and *Agathobacter* showed a decreasing trend in CON compared with ML (Figure 4D). Correlation analysis between differential bacteria and lipids showed that UCG−005, Muribaculaceae_norank, Prevotellaceae_NK3B31_group, *Prevotella*, Rumi-nococcaceae_uncultured, *Limosilactobacillus*, and *Monoglobus* were significantly positively correlated with IMF and TG (Figure 4E). The correlation analysis between differential bacteria and acyl groups in TG showed that UCG−005, Muribaculaceae_norank, Prevotellaceae_NK3B31_group, and Ruminococcaceae_uncultured were positively correlated with the precursor of linoleic acid (C18:2) and negatively correlated with oleic acid precursors (C18:1) (Figure 4F). These results suggest that mulberry leaf supplementation may regulate IMF by improving the relative abundances of UCG−005, *Muribaculaceae*_norank, *Prevotellaceae*_NK3B31_group, and *Ruminococcaceae*_uncultured.

### 3.4. Untargeted Metabolomics Analysis of Cecal Contents

**Figure 5:**
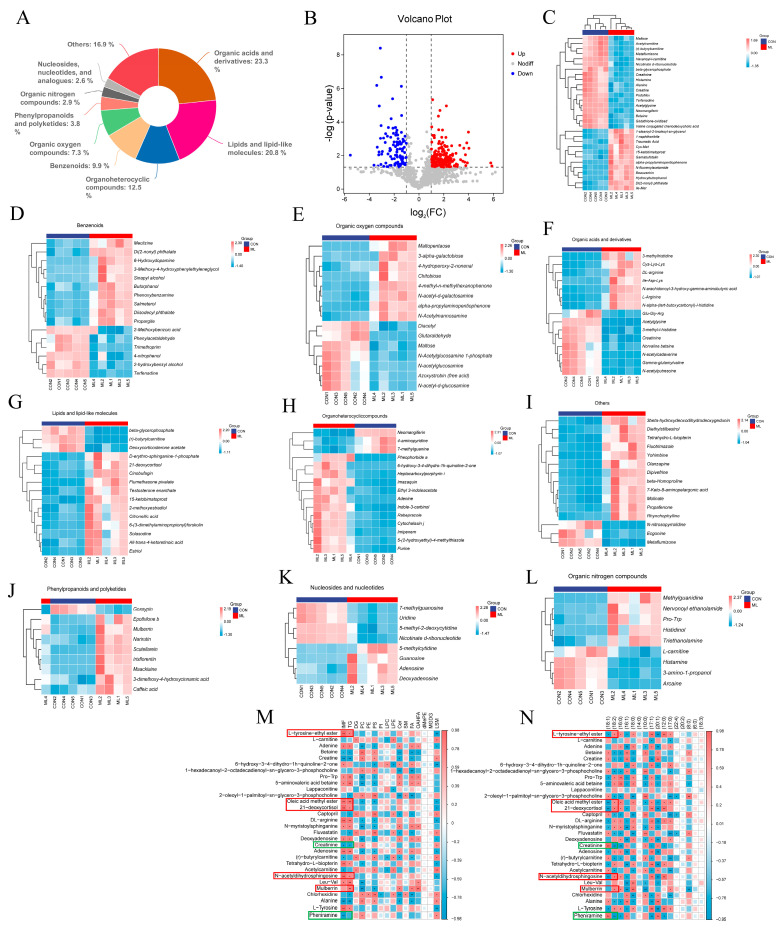
Effects of mulberry leaves on cecal metabolites. (**A**) Proportional composition of cecal metabolites. (**B**) Volcano plot of cecal metabolites. (**C**) Heat map of the top 30 differentially expressed metabolites. (**D**–**L**) Heat map of various differential cecal metabolites. (**M**) Heat map of the correlation between differential cecal metabolites and lipid species. (**N**) Heat map of the correlation between differential cecal metabolites and TG acyl groups. * *p* < 0.05 and ** *p* < 0.01.

To explore the effects of mulberry leaves on cecal contents, an untargeted metabolomic analysis was performed. A pie chart is shown to describe the proportion of various compounds identified in the contents of the cecum; the top three compounds were organic acids and their derivatives, lipids and lipid-like molecules, and organic heterocyclic compounds (Figure 5A). We screened 338 differential compounds (*p* < 0.05, FC > 2) and generated a volcano plot to visualize significant differences (Figure 5B). Heat maps of the top 30 differentially expressed compounds were plotted. The expression levels of maltose, acetylcarnitine, and creatine were significantly downregulated, whereas the expression levels of 1-stearoyl-2-linoleoyl-sn-glycerol and traumatic acid were significantly upregulated (Figure 5C). Subsequently, the differential compounds were classified, and heat maps were drawn sequentially (Figure 5D–L). The correlation analysis between different compounds and IMF and lipid species revealed that L-tyrosine-ethyl ester, oleic acid methyl ester, 21-deoxycortisol, N-acetyldihydrosphingosine, and mulberrin were positively correlated with TG, while creatinine and pheniramine were negatively correlated with TG (Figure 5M). Correlation analysis between different compounds and the acyl groups of TG showed that L-tyrosine-ethyl ester, oleic acid methyl ester, 21−deoxycortisol, N-N-acetyldihydrosphingosine, and mulberrin were significantly positively correlated with the precursor of linoleic acid (C18:2) and negatively correlated with oleic acid precursors (C18:1) (Figure 5N). These results suggest that mulberry leaves may affect IMF content through the regulation of cecal L-tyrosine-ethyl ester, oleic acid methyl ester, 21-deoxycortisol, N-acetyldihydrosphingosine, and mulberrin.

### 3.5. Expression of Genes Involved in Lipid Metabolism and Changes in Regulatory Factors in Serum

**Figure 6:**
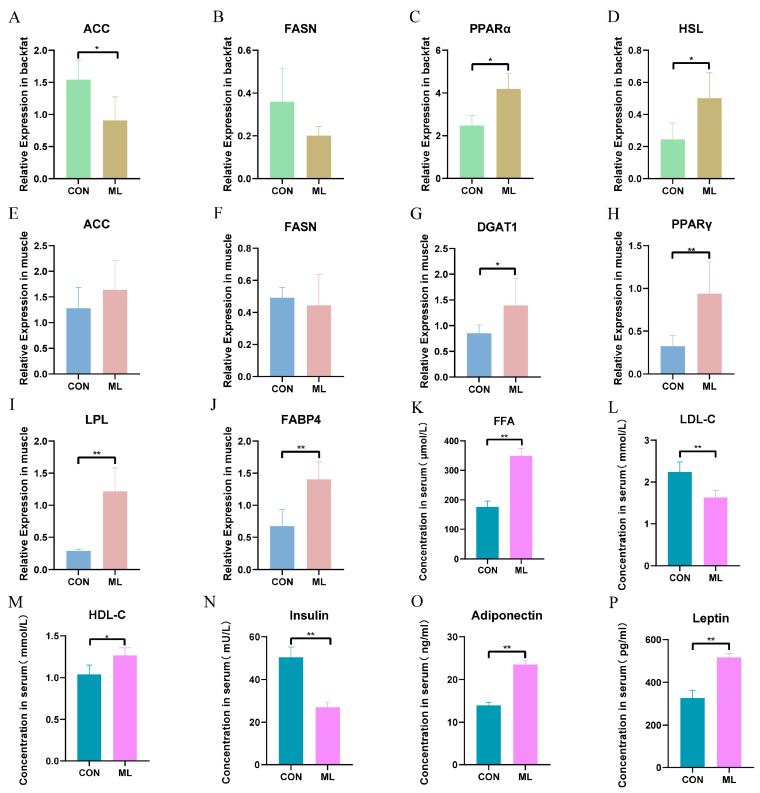
Effect of mulberry leaves on the expression of key genes involved in lipid metabolism and on serum indicators of lipid metabolism. (**A**–**D**) Effect of mulberry leaves on the relative expression of ACC, FASN, PPARα, and HSL in backfat. (**E**–**J**) Effect of ML supplementation on the relative expression of ACC, FASN, DGAT1, PPARγ, LPL, and FABP4 in the muscle. (**K**–**P**) Effect of ML supplementation on serum FFA, LDL-C, HDL-C, insulin, adiponectin, and leptin. * *p* < 0.05 and ** *p* < 0.01.

To further explore the mechanism by which mulberry leaves improve meat quality, we measured the regulation of genes related to lipid metabolism alongside several serum indicators. These included genes related to fatty acid synthesis such as acetyl CoA carboxylase (ACC) and fatty acid synthase (FASN), genes related to fatty acid transport such as lipoprotein lipase (LPL) and fatty acid-binding protein 4 (FABP4), genes related to triglyceride synthesis such as diacylglycerol acyltransferase 1 (DGAT1), genes related to triglyceride breakdown such as hormone-sensitive triglyceride lipase (HSL), and key genes for prelipid cell differentiation such as peroxisome proliferator-activated receptor γ (PPARγ). The expression of ACC was significantly downregulated in backfat (*p* < 0.05), whereas the expression levels of peroxisome proliferator-activated receptor α (PPARα) and HSL were significantly upregulated (*p* < 0.05) (Figure 6A–D). The expression levels of FABP4, LPL, DGAT1, and PPARγ were significantly upregulated in muscle tissue (*p* < 0.01), whereas the expression levels of ACC and FASN were not significantly different (Figure 6E–J). Serum indices revealed that the expression of low-density lipoprotein cholesterol (LDL-C) and insulin was significantly downregulated (*p* < 0.01), the expression of high-density lipoprotein cholesterol (HDL-C) was significantly upregulated (*p* < 0.05), and the expression of leptin, adiponectin, and free fatty acid was significantly upregulated (*p* < 0.01) (Figure 6K–P). These results indicate that ML supplementation may inhibit backfat deposition by inhibiting the relative expression of ACC while promoting PPARα and HSL expression. ML may also increase IMF by promoting FABP4, LPL, and PPARγ expression in the muscle. The increase in serum leptin and adiponectin levels may also be one of the factors regulating lipid deposition in muscle and backfat.

## 4. Discussion

In this study, we found that dietary supplementation with mulberry leaves significantly improved the meat quality of pork. Mulberry leaves are often used in traditional Chinese medicine to reduce blood glucose and improve lipid levels [10]. Greater attention has been paid to their application in non-alcoholic hepatitis and dyslipidemia, so their potential to improve animal meat quality has been understudied. Related studies have found that mulberry leaf supplementation can enhance the nutritional value of pork. Long-term supplementation with mulberry leaves has been associated with an increase in the protein content and essential amino acids in meat, which are crucial for muscle growth and meat quality [12]. Related studies have found that there are a variety of antioxidants in mulberry leaves, among which the flavonoids of mulberry leaves can effectively reduce lipid oxidation during the storage and transportation of meat, as well as maintain the color and taste of meat [13]. This aspect is particularly important from a marketability standpoint, as consumer preference often leans towards meat products with superior taste and aroma. Backfat thickness is an important indicator of performance and meat quality; excess backfat frequently has a negative effect on the reproductive performance, immunity, and meat quality of pigs [24,25]. There are many factors affecting backfat, including genetic factors, nutrition, gender, and environment, among which genetics is the primary factor [26]. Some breeds have backfat thicknesses ranging from 35 to 45 mm. For example, the backfat thickness of Guanzhong black pigs can reach 40 mm at 10 months of age, and the backfat thickness of Wulian black pigs is 38–40 mm at 110 kg [27,28]. For some breeds with lower backfat thickness, such as Chuanxiang black pigs, the thickness is 18–20 mm at 100–110 kg; Large White pigs have a thickness of 17–18 mm at 100 kg; and Berkshire pigs have a thickness of 21–28 mm at 120 kg [29,30,31]. Reducing backfat is key to improving the ratio of protein to fat, thereby increasing the lean meat rating. Studies have found that mulberry leaves can inhibit the deposition of backfat by promoting the secretion of leptin and the expression of the lipolysis gene HSL in the backfat tissue of pigs while inhibiting the expression of the fatty acid synthesis gene FASN, which is consistent with our results [16]. Leptin, a cytokine secreted by adipocytes, can promote lipolysis and inhibit fat deposition, which may be related to the regulation of backfat by mulberry leaves [32]. In addition, mulberry leaves may regulate fat deposition through the de novo fatty acid synthesis (DNL) pathway. ACC and FASN are the key genes upstream of the DNL pathway; we found that mulberry leaf supplementation had a significant inhibitory effect on ACC in backfat tissue, which may be causative for the inhibition of backfat deposition we observed in the ML group [33,34]. Previous research has shown that MLPs may increase the activity of brown adipocytes, accelerate fat consumption, and inhibit fat deposition, which may be one of the mechanisms by which MLP inhibits backfat deposition [35]. In addition, MLP can inhibit the expression of pancreatic lipase and reduce the intestinal absorption ability of dietary fat, thus playing an anti-obesity role [36].

IMF is an important component determining meat tenderness. Factors that affect fat deposition include genetics, nutrition, age, and environment [37,38]. While there are known site-dependent differences, the specific mechanism underlying fat deposition remains unclear. Backfat and IMF content are important indicators of meat quality. A higher IMF content, higher marbling score, and lower backfat content are often preferred by consumers. Unfortunately, backfat and IMF often increase correlatively, so it is very difficult to find an ideal balance between IMF and backfat. Improving meat quality through dietary modification represents the mainstream strategy for achieving this balance. Previous studies have found that mulberry leaves may promote IMF deposition and improve amino acid composition, which is consistent with our results; however, its specific mechanism of action remains unclear [13]. It has been shown that flavonoids in mulberry leaf extract can significantly stimulate the differentiation of 3T3-L1 cells and promote adiponectin secretion, which may in turn promote IMF deposition [39]. We additionally found that ML supplementation significantly increased the serum levels of adiponectin and leptin, two cytokines secreted during adipocyte differentiation, while promoting the expression of PPARγ and inhibiting insulin resistance [40]. This difference in IMF deposition may be related to the regulation of adiponectin levels in mulberry leaves. We found that ML supplementation significantly increased the expression of LPL, FABP4, and DGAT1, which are key genes involved in cellular fatty acid uptake and the rate-limiting gene for triglyceride synthesis [41,42]. These results suggest that mulberry leaves may promote fatty acid uptake and TG synthesis by upregulating the expression of LPL, FABP4, and DGAT1, thereby increasing IMF.

Lipidomics and metabolomics have played an important role in exploring microscopic changes in lipids and metabolites; these analyses have been used to identify potential biomarkers and trace upstream pathways through the deep correlation of differentiators, which can help in elucidating the underlying mechanisms [43,44,45]. Here, we used lipidomics to analyze differences in lipid molecules in muscle and identified four major differential molecules: TG, PE, PC, and FA. We additionally discovered that dietary supplementation with mulberry leaves enhanced muscle C18:2 content, which was significantly positively correlated with IMF. Linoleic acid, a precursor of the C18:2 acyl group in lipids, may be associated with IMF; conjugated linoleic acid, a heterodimer of linoleic acid, has been reported to increase the deposition of IMF in animals through activation of the PPARα/FAPB4 signaling pathway [46]. Likewise, the C18:0 precursor palmitic acid was also positively correlated with IMF. It has been reported that palmitic acid can induce endoplasmic reticulum stress through activation of the NF-κB signaling pathway, which in turn leads to the deposition of lipid tissues and promotes the release of exosome miR-4431. Our findings suggest that mulberry leaves may improve IMF deposition by increasing linoleic acid and palmitic acid [47,48].

Prior research has demonstrated that the addition of 10% ML content to a standard diet significantly increased the C18:2 content of free fatty acids in muscle while reducing the C18:1 content, which supports our findings regarding the acyl group of muscle TG [49]. There are two possible reasons for this finding: the higher cellular uptake of linoleic acid compared to oleic acid results in a higher C18:2 ratio and a lower C18:1 ratio in TG, or as IMF increases, fatty acids synthesized by the DNL pathway or taken up from the blood circulation are more likely to be converted into linoleic acid rather than oleic acid in cells. Studies have shown that fatty acids are more inclined to be converted into long-chain polyunsaturated fatty acids in mature 3T3-L1 cells, but the underlying mechanism remains unclear [50]. Analysis of meat quality often focuses on exploring the distribution and differences of free fatty acids in muscle. However, the content of free fatty acids in muscle is much lower than that of TG, and the composition of free fatty acids is unstable and readily influenced by a variety of factors; therefore, the analysis of the TG acyl chain is more intuitive.

Mulberry leaves have been shown to exert positive effects on gut health, with research indicating that they can increase the abundance of Bacteroidetes and Proteobacteria, while reducing the abundance of Firmicutes and Bacteroidetes [51]. Previous studies have demonstrated that mulberry leaves can improve the gut microbiota and glucose homeostasis in rats with type 2 diabetes mellitus (T2DM) [52]. Furthermore, mulberry leaves have been proven to restore the abundance of Bacteroidetes and Proteobacteria, which are highly associated with diabetes, and to improve lipid homeostasis by inhibiting the NEFA pathway [53]. The cecal microbiota is a central factor in the regulation of lipid metabolism and host immunity. However, the effect of mulberry leaves on the gut microbiota and the regulatory mechanisms between gut microbiota and lipids remain unclear. Firmicutes and Bacteroidetes are the main phyla of microorganisms colonizing the mammalian gut, and studies have found that the ratio of Firmicutes to Bacteroidetes is positively correlated with obesity [54,55]. Mulberry leaves significantly increased the abundance of Bacteroidetes in the cecum, leading to a decrease in the ratio of Firmicutes to Bacteroidetes, which may be related to their anti-obesity effect. We found that UCG−005, *Muribaculaceae*_norank, *Prevotellaceae*_NK3B31_group, *Prevotella*, *Limosilactobacillus*, *Ruminococcaceae*_uncultured, and *Monoglobus* were significantly positively correlated with IMF and TG. Among these, UCG−005, *Muribaculaceae*_norank, *Prevotellaceae*_NK3B31_group, and *Ruminococcaceae*_uncultured were positively correlated with the precursor of linoleic acid (C18:2) and negatively correlated with the oleic acid precursor (C18:1). It has been reported that UCG−005 is correlated with circulating acetate [56], and *Prevotellaceae*_NK3B31_group is often regarded as the main producer of butyrate [57]. Short-chain fatty acids are closely related to meat quality and lipid metabolism, suggesting that mulberry leaves may regulate lipid deposition in pig muscle by changing the composition of the bacterial flora [58]. Within the cecal contents, mulberrin and oleic acid methyl ester correlated positively with IMF and negatively with oleate. Gut metabolites are closely related to multiple physiological processes and metabolic pathways in the host [59]. Here, a variety of gut-differentiated compounds were found to be associated with IMF, among which mulberry leaf flavonoids are most noteworthy because mulberry-leaf-derived compounds and flavonoids are closely related to lipid metabolism. Flavonoids in mulberry leaves have been reported to promote adipocyte differentiation by increasing the expression of PPARγ and FABP4 genes, which act as candidate genes for the regulation of IMF. Our findings suggest that flavonoids in fermented mulberry leaves may act as potential modulators that regulate IMF deposition by promoting the expression of FABP4 [39]. Oleic acid methyl ester may act as a precursor of C18:2 after intestinal uptake into the blood circulation; however, the specific mechanism remains a topic for future research.

## 5. Conclusions

Feeding Yuxi black pigs an 8% mulberry leaf diet may reduce backfat and increase intramuscular fat, which we speculate may be associated with the downregulation of ACC and FASN in the DNL pathway, as well as the upregulation of LPL, FABP4, and DGAT1. Concurrently, we observed that the ratio of C18:1 and C18:2 might be closely related to fat deposition. The specific mechanisms of these hypothesized regulatory pathways are still to be explored. Mulberry leaves may also influence pork fat deposition through the regulation of gut microbiota and intestinal metabolites. This work lays the foundation for further investigation into the mechanisms by which mulberry leaves and their components affect meat quality.

## Figures and Tables

**Table 1:** Growth performance of Yuxi black pigs.

	CON ^1^	ML ^1^	SEM ^2^	*p*-Value
Initial weight	41.2 ± 2.96	42.1 ± 3.29	1.14	0.42
Final weight	112.3 ± 8.01	111.5 ± 7.56	2.844	0.77
ADG (kg/d)	0.59 ± 0.06	0.58 ± 0.08	0.026	0.57
ADFI (kg/d)	2.18 ± 0.15	2.12 ± 0.14	0.05	0.25
F:G ratio	3.71 ± 0.39	3.74 ± 0.63	0.19	0.86

^1^ CON, control group; ML, mulberry leaf group. ^2^ SEM, standard error of the mean. Results are presented as mean ± SD, *n* = 15. ADG, average daily gain; ADFI, average daily feed intake; F:G ratio, ratio of feed to gain. SEM, standard error of the mean.

**Table 2:** Effects of mulberry leaves on the meat quality of Yuxi pigs ^1^.

Meat Quality Traits	CON ^1^	ML ^1^	SEM ^3^	*p*-Value
Lightness (L*)	49.90 ± 3.35	54.31 ± 3.06 **	1.17	0.001
Redness (a*)	13.73 ± 1.42	13.71 ± 1.64	0.56	0.98
Yellowness (b*)	6.03 ± 0.99	5.67 ± 1.26	0.41	0.38
Marbling score ^2^	3.72 ± 0.63	4.23 ± 0.36 *	0.19	0.011
pH (45 min)	6.46 ± 0.28	6.38 ± 0.32	0.11	0.46
IMF content (%)	3.44 ± 0.43	4.18 ± 0.56 **	0.18	0.0004
Muscle TG (mg/g)	13.09 ± 2.11	20.29 ± 2.89 **	0.92	0.001
Shear force	19.23 ± 3.41	19.96 ± 3.93	1.34	0.59
Backfat thickness (mm)	24.60 ± 4.12	20.73 ± 4.10 *	1.50	0.016
Backfat TG (mg/g)	638.6 ± 54.62	558.1 ± 35.33 **	16.80	0.001

^1^ CON, control group; ML, mulberry leaf group; ^2^ Marbling score: 1 = trace marbling and 5 = extremely rich marbling. ^3^ SEM, standard error of the mean. Results are presented as mean ± SD, *n* = 15. * *p* < 0.05 and ** *p* < 0.01.

## Data Availability

The data are available on request.

## Supplementary Materials

The following supporting information can be downloaded at https://www.mdpi.com/article/10.3390/ani14081233/s1. Figure S1: Species rarefaction curve. Figure S2: The index of Chao1 Shannon Simpson. Table S1: The nutritional composition of the daily diet. Table S2: The primer sequence of qPCR.

## References

[1] González-García S., Esteve-Llorens X., Moreira M.T., Feijoo G. (2018). Carbon footprint and nutritional quality of different human dietary choices. Sci. Total Environ..

[2] Reig M., Aristoy M.C., Toldrá F. (2013). Variability in the contents of pork meat nutrients and how it may affect food composition databases. Food Chem..

[3] Collins L.M., Smith L.M. (2022). Review: Smart agri-systems for the pig industry. Animal.

[4] Kong C., Yang L., Gong H., Wang L., Li H., Li Y., Wei B., Nima C., Deji Y., Zhao S. (2022). Dietary and Food Consumption Patterns and Their Associated Factors in the Tibetan Plateau Population: Results from 73 Counties with Agriculture and Animal Husbandry in Tibet, China. Nutrients.

[5] Malgwi I.H., Halas V., Grünvald P., Schiavon S., Jócsák I. (2022). Genes Related to Fat Metabolism in Pigs and Intramuscular Fat Content of Pork: A Focus on Nutrigenetics and Nutrigenomics. Animals.

[6] Fortin A., Robertson W.M., Tong A.K. (2005). The eating quality of Canadian pork and its relationship with intramuscular fat. Meat Sci..

[7] Zhang Y., Sun Y., Wu Z., Xiong X., Zhang J., Ma J., Xiao S., Huang L., Yang B. (2021). Subcutaneous and intramuscular fat transcriptomes show large differences in network organization and associations with adipose traits in pigs. Sci. China Life Sci..

[8] Santos-Silva J., Francisco A., Portugal A.P., Paulos K., Dentinho M.T., Almeida J.M., Regedor L., Fialho L., Cachucho L., Jerónimo E. (2022). Effects of partial substitution of grain by agroindustrial byproducts and sunflower seed supplementation in beef haylage-based finisher diets on growth, in vitro methane production and carcass and meat quality. Meat Sci..

[9] Fixen P.E., Johnston A.M. (2012). World fertilizer nutrient reserves: A view to the future. J. Sci. Food Agric..

[10] Zhang R., Zhang Q., Zhu S., Liu B., Liu F., Xu Y. (2022). Mulberry leaf (*Morus alba L*.): A review of its potential influences in mechanisms of action on metabolic diseases. Pharmacol. Res..

[11] Ma G., Chai X., Hou G., Zhao F., Meng Q. (2022). Phytochemistry, bioactivities and future prospects of mulberry leaves: A review. Food Chem..

[12] Liu Y., Li Y., Peng Y., He J., Xiao D., Chen C., Li F., Huang R., Yin Y. (2019). Dietary mulberry leaf powder affects growth performance, carcass traits and meat quality in finishing pigs. J. Anim. Physiol. Anim. Nutr..

[13] Liu Y., Li Y., Xiao Y., Peng Y., He J., Chen C., Xiao D., Yin Y., Li F. (2021). Mulberry leaf powder regulates antioxidative capacity and lipid metabolism in finishing pigs. Anim. Nutr..

[14] Parida I.S., Takasu S., Nakagawa K. (2023). A comprehensive review on the production, pharmacokinetics and health benefits of mulberry leaf iminosugars: Main focus on 1-deoxynojirimycin, d-fagomine, and 2-O-α-d-galactopyranosyl-DNJ. Crit. Rev. Food Sci. Nutr..

[15] Cui W., Luo K., Xiao Q., Sun Z., Wang Y., Cui C., Chen F., Xu B., Shen W., Wan F. (2023). Effect of mulberry leaf or mulberry leaf extract on glycemic traits: A systematic review and meta-analysis. Food Funct..

[16] Fan L., Peng Y., Wu D., Hu J., Shi X., Yang G., Li X. (2020). Dietary supplementation of Morus nigra L. leaves decrease fat mass partially through elevating leptin-stimulated lipolysis in pig model. J. Ethnopharmacol..

[17] Milani C., Duranti S., Bottacini F., Casey E., Turroni F., Mahony J., Belzer C., Delgado Palacio S., Arboleya Montes S., Mancabelli L. (2017). The First Microbial Colonizers of the Human Gut: Composition, Activities, and Health Implications of the Infant Gut Microbiota. Microbiol. Mol. Biol. Rev..

[18] Edwards B.R. (2023). Lipid Biogeochemistry and Modern Lipidomic Techniques. Ann. Rev. Mar. Sci..

[19] Ma X., Zhang W., Li Z., Xia Y., Ouyang Z. (2021). Enabling High Structural Specificity to Lipidomics by Coupling Photochemical Derivatization with Tandem Mass Spectrometry. Acc. Chem. Res..

[20] Hillesheim E., Brennan L. (2023). Distinct patterns of personalised dietary advice delivered by a metabotype framework similarly improve dietary quality and metabolic health parameters: Secondary analysis of a randomised controlled trial. Front. Nutr..

[21] Rao X., Huang X., Zhou Z., Lin X. (2013). An improvement of the 2ˆ(-delta delta CT) method for quantitative real-time polymerase chain reaction data analysis. Biostat. Bioinform. Biomath..

[22] Liu W., Chen L., Miao K., You Y., Li J., Lu J., Zhang Y. (2023). Identification and validation of diagnostic biomarkers for intrahepatic cholestasis of pregnancy based on untargeted and targeted metabolomics analyses of urine metabolite profiles. BMC Pregnancy Childbirth.

[23] Wang H., Meng L., Mi L. (2023). Effects of Leymus chinensis hay and alfalfa hay on growth performance, rumen microbiota, and untargeted metabolomics of meat in lambs. Front. Vet. Sci..

[24] Gilbert H., Billon Y., Brossard L., Faure J., Gatellier P., Gondret F., Labussière E., Lebret B., Lefaucheur L., Le Floch N. (2017). Review: Divergent selection for residual feed intake in the growing pig. Animal.

[25] Li X., Xiong X., Wu X., Liu G., Zhou K., Yin Y. (2020). Effects of stocking density on growth performance, blood parameters and immunity of growing pigs. Anim. Nutr..

[26] Gozalo-Marcilla M., Buntjer J., Johnsson M., Batista L., Diez F., Werner C.R., Chen C.Y., Gorjanc G., Mellanby R.J., Hickey J.M. (2021). Genetic architecture and major genes for backfat thickness in pig lines of diverse genetic backgrounds. Genet. Sel. Evol..

[27] Cai R., Chao M., Zhao T., Li R., Zhang Z., Yan W., Pang W. (2023). miR-503 targets MafK to inhibit subcutaneous preadipocyte adipogenesis causing a decrease of backfat thickness in Guanzhong Black pigs. Meat Sci..

[28] Guo J.F. (2022). Comparison of carcass performance and meat quality of Wulian black pigs with different slaughter weights. Chin. J. Pig Farming Today.

[29] Yang X., Gu Y., Yang Y., Liang Y., Tao X., Zhong Z., Zeng K., Chen X., Gong J., Lei Y. (2021). Breeding of Chuanxiang black pigs. Chin. J. Anim. Sci..

[30] Lu Y., Yao H., Zeng T., Lei T., Shi J. (2017). A comparative study on the difference of backfat thickness between Nanyang black pig and large white pig in Qi Changcheng. Chin. J. Pig Breed..

[31] Ohkoda T., Yoshida K., Ijiri D., Ohtsuka A. (2021). Effect of mixed rearing of barrows and gilts on backfat thickness and serum metabolite profiles of the Kagoshima-Kurobuta (Berkshire) pig. Anim. Sci. J..

[32] Li Q., Liao S., Pang D., Li E., Liu T., Liu F., Zou Y. (2022). The transported active mulberry leaf phenolics inhibited adipogenesis through PPAR-γ and Leptin signaling pathway. J. Food Biochem..

[33] He L., Xing Y., Ren X., Zheng M., Yu S., Wang Y., Xiu Z., Dong Y. (2022). Mulberry Leaf Extract Improves Metabolic Syndrome by Alleviating Lipid Accumulation In Vitro and In Vivo. Molecules.

[34] Qin L., Huang T., Jing R., Wen J., Cao M. (2023). Mulberry leaf extract reduces abdominal fat deposition via adenosine-activated protein kinase/sterol regulatory element binding protein-1c/acetyl-CoA carboxylase signaling pathway in female Arbor Acre broilers. Poult. Sci..

[35] Li R., Xue Z., Li S., Zhou J., Liu J., Zhang M., Panichayupakaranant P., Chen H. (2022). Mulberry leaf polysaccharides ameliorate obesity through activation of brown adipose tissue and modulation of the gut microbiota in high-fat diet fed mice. Food Funct..

[36] Li R., Xue Z., Jia Y., Wang Y., Li S., Zhou J., Liu J., Zhang M., He C., Chen H. (2021). Polysaccharides from mulberry (*Morus alba* L.) leaf prevents obesity by inhibiting pancreatic lipase in high-fat diet induced mice. Int. J. Biol. Macromol..

[37] Cesar A.S., Regitano L.C., Koltes J.E., Fritz-Waters E.R., Lanna D.P., Gasparin G., Mourão G.B., Oliveira P.S., Reecy J.M., Coutinho L.L. (2015). Putative regulatory factors associated with intramuscular fat content. PLoS ONE.

[38] Moisá S.J., Shike D.W., Faulkner D.B., Meteer W.T., Keisler D., Loor J.J. (2014). Central Role of the PPARγ Gene Network in Coordinating Beef Cattle Intramuscular Adipogenesis in Response to Weaning Age and Nutrition. Gene Regul. Syst. Bio.

[39] Naowaboot J., Chung C.H., Pannangpetch P., Choi R., Kim B.H., Lee M.Y., Kukongviriyapan U. (2012). Mulberry leaf extract increases adiponectin in murine 3T3-L1 adipocytes. Nutr. Res..

[40] Yang W., Yuan W., Peng X., Wang M., Xiao J., Wu C., Luo L. (2019). PPAR γ/Nnat/NF-κB Axis Involved in Promoting Effects of Adiponectin on Preadipocyte Differentiation. Mediat. Inflamm..

[41] Boss M., Kemmerer M., Brüne B., Namgaladze D. (2015). FABP4 inhibition suppresses PPARγ activity and VLDL-induced foam cell formation in IL-4-polarized human macrophages. Atherosclerosis.

[42] Yin B.Z., Fang J.C., Zhang J.S., Zhang L.M., Xu C., Xu H.Y., Shao J., Xia G.J. (2020). Correlations between single nucleotide polymorphisms in FABP4 and meat quality and lipid metabolism gene expression in Yanbian yellow cattle. PLoS ONE.

[43] Han X., Gross R.W. (2022). The foundations and development of lipidomics. J. Lipid Res..

[44] Khanna R.K., Catanese S., Emond P., Corcia P., Blasco H., Pisella P.J. (2022). Metabolomics and lipidomics approaches in human tears: A systematic review. Surv. Ophthalmol..

[45] Wang R., Li B., Lam S.M., Shui G. (2020). Integration of lipidomics and metabolomics for in-depth understanding of cellular mechanism and disease progression. J. Genet. Genom..

[46] Chen J., You R., Lv Y., Liu H., Yang G. (2022). Conjugated linoleic acid regulates adipocyte fatty acid binding protein expression via peroxisome proliferator-activated receptor α signaling pathway and increases intramuscular fat content. Front. Nutr..

[47] Li X., Mai K., Ai Q. (2023). Palmitic acid activates NLRP3 inflammasome through NF-κB and AMPK-mitophagy-ROS pathways to induce IL-1β production in large yellow croaker (*Larimichthys crocea*). Biochim. Biophys. Acta Mol. Cell Biol. Lipids.

[48] Wan S.R., Teng F.Y., Fan W., Xu B.T., Li X.Y., Tan X.Z., Guo M., Gao C.L., Zhang C.X., Jiang Z.Z. (2023). BDH1-mediated βOHB metabolism ameliorates diabetic kidney disease by activation of NRF2-mediated antioxidative pathway. Aging.

[49] Niu J., Liu X., Xu J., Li F., Wang J., Zhang X., Yang X., Wang L., Ma S., Li D. (2023). Effects of Silage Diet on Meat Quality through Shaping Gut Microbiota in Finishing Pigs. Microbiol. Spectr..

[50] Wunderling K., Zurkovic J., Zink F., Kuerschner L., Thiele C. (2023). Triglyceride cycling enables modification of stored fatty acids. Nat. Metab..

[51] Du Y., Li D., Lu Y., Zhang R., Zheng X., Xu B., Zhao Y., Ji S., Guo M., Wang L. (2022). *Morus alba* L. water extract changes gut microbiota and fecal metabolome in mice induced by high-fat and high-sucrose diet plus low-dose streptozotocin. Phytother. Res..

[52] Liu Z., Liu Q., Liu Z., Tang J., Chua E., Li F., Xiong X., Wang M., Wen P., Shi X. (2021). Ethanol extract of mulberry leaves partially restores the composition of intestinal microbiota and strengthens liver glycogen fragility in type 2 diabetic rats. BMC Complement. Med. Ther..

[53] Sheng Y., Zheng S., Ma T., Zhang C., Ou X., He X., Xu W., Huang K. (2017). Mulberry leaf alleviates streptozotocin-induced diabetic rats by attenuating NEFA signaling and modulating intestinal microflora. Sci. Rep..

[54] Magne F., Gotteland M., Gauthier L., Zazueta A., Pesoa S., Navarrete P., Balamurugan R. (2020). The Firmicutes/Bacteroidetes Ratio: A Relevant Marker of Gut Dysbiosis in Obese Patients?. Nutrients.

[55] Stojanov S., Berlec A., Štrukelj B. (2020). The Influence of Probiotics on the Firmicutes/Bacteroidetes Ratio in the Treatment of Obesity and Inflammatory Bowel disease. Microorganisms.

[56] Shen C.L., Wang R., Ji G., Elmassry M.M., Zabet-Moghaddam M., Vellers H., Hamood A.N., Gong X., Mirzaei P., Sang S. (2022). Dietary supplementation of gingerols- and shogaols-enriched ginger root extract attenuate pain-associated behaviors while modulating gut microbiota and metabolites in rats with spinal nerve ligation. J. Nutr. Biochem..

[57] Li Q., Cao M., Wei Z., Mei J., Zhang Y., Li M., Li M., Zhang Y., Wang Z. (2022). The protective effect of Buzhong Yiqi decoction on ischemic stroke mice and the mechanism of gut microbiota. Front. Neurosci..

[58] Zhang H., Qin S., Zhang X., Du P., Zhu Y., Huang Y., Michiels J., Zeng Q., Chen W. (2022). Dietary resistant starch alleviates Escherichia coli-induced bone loss in meat ducks by promoting short-chain fatty acid production and inhibiting Malt1/NF-κB inflammasome activation. J. Anim. Sci. Biotechnol..

[59] Krishnamurthy H.K., Pereira M., Bosco J., George J., Jayaraman V., Krishna K., Wang T., Bei K., Rajasekaran J.J. (2023). Gut commensals and their metabolites in health and disease. Front. Microbiol..

