# Supplementary material for: Mulberry Leaf Dietary Supplementation Can Improve the Lipo-Nutritional Quality of Pork and Regulate Gut Microbiota in Pigs: A Comprehensive Multi-Omics Analysis
Source: Animals (Basel). 2024 Apr 19;14(8):1233. doi: 10.3390/ani14081233 (PMC11047539; doi:10.3390/ani14081233)

Supplementary Material

Supplementary Figures and Tables

Supplementary Tables

Supplementary Table S1. The nutritional composition of the daily diet

Diet composition (%)	CON group	ML group
Mulberry leaves	0.00	8.00
Soybean meal	14.00	14.00
Puffing of soybean	5.00	5.00
Soy protein concentrate	1.00	1.00
Wheat bran	7.00	0.00
corn	61.00	60.60
Protect merchant	0.30	0.30
Fish meal	0.00	0.00
A powder	6.00	5.00
salt	0.40	0.40
Soybean oil	1.00	1.00
montmorillonite	0.20	0.20
In the pig premix	2.00	2.00
Stone powder	1.20	1.20
Calcium hydrogen	1.00	1.40

A combined	100.10	100.10
Total digestible energy (MJ/kg)	13.85	13.60
Crude protein (%)	15.60	15.66
Lysine (%)	0.94	0.93
(egg + cysteine) (%)	0.65	0.63
Threonine (%)	0.52	0.50
Tryptophan (%)	0.22	0.21
Calcium (%)	0.82	0.85
Total phosphorus (%)	0.54	0.55

Note: per kg of premix to feed: Fe,155mg/kg diet; Cu,200mg; n 140mg; Mn,50mg; VA,17500IU; VE,20IU; Riboflavin, 3.6mg; Niacin, 27mg; Pantothenic acid, 13mg; B12, 0.09 mg; Biotin, 0.15mg; Choline, 0.92mg

Supplementary Table S2 The primer sequence of qPCR

Primer name	Sequence (5'-3')
ACC-F	GGCCATCAAGGACTTCAACC
ACC-R	ACGATGTAAGCGCCGAACTT
FASN-F	GTCCTGCTGAAGCCTAACTC
FASN-R	TCCTTGGAACCGTCTGTG
PPARγ-F	AGCCCTTTGGTGACTT
PPARγ-R	AGGACTCTGGGTGGTT

FABP4-F	CTGAGATTGCCTTCAAATTG
FABP4-R	CTTGGCTTATGCTCTCTCATA
LPL-F	CTCGTGCTCAGATGCCCTAC
LPL-R	GGCAGGGTGAAAGGGATGTT
PPARα-F	AGAGCCCCATCTGTCCTCTC
PPARα-R	ACTGGTAGTCTGCAAAACCAAA
HSL-F	CTTTCGCACCAGCCACAAC
HSL-R	CTCGTCGCCCTCAAAGAAGA
DGAT1-F	TGGACTACTCACGCATCAT
DGAT1-R	GTGGAAGAGCCAGTAGAAGAA

Supplementary Figure

Supplementary Figure S1 Species rarefaction curve

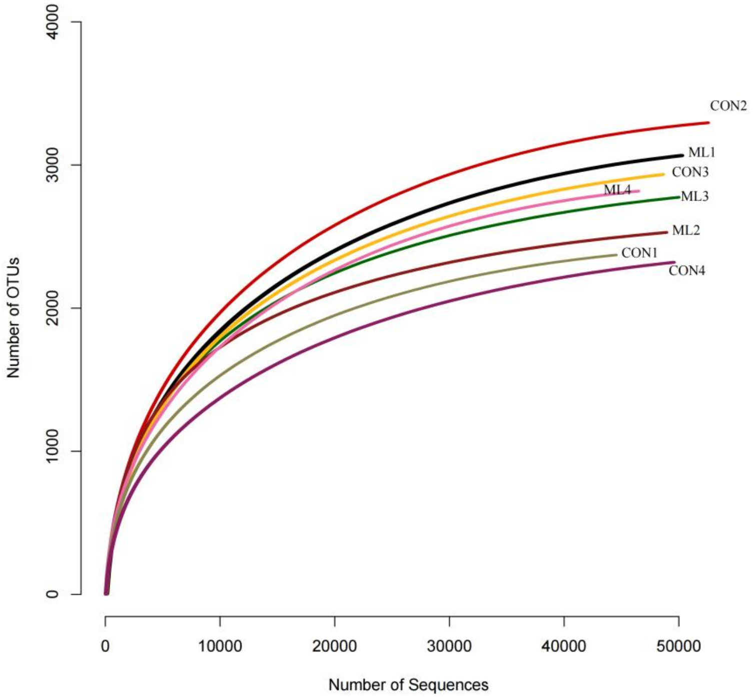

Supplementary Figure S2 The index of Chao1 Shannon Simpson

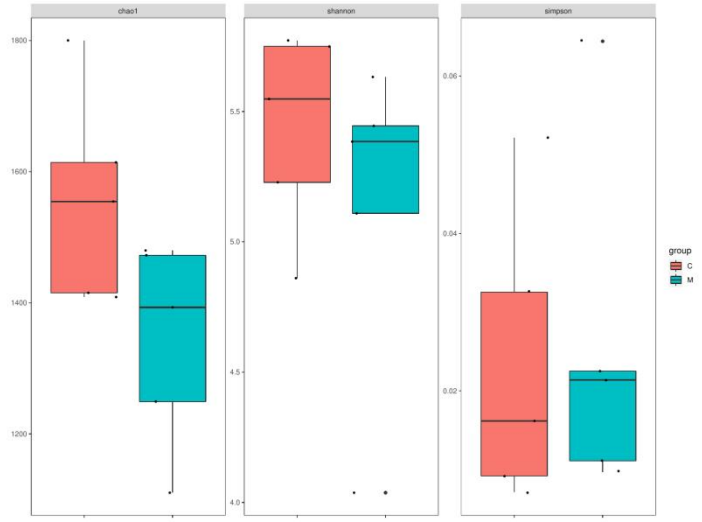

Supplement: Supplementary file 1 [file animals-14-01233-s001.zip › animals-2914589-supplementary.pdf]
